# 2307: Resting state network profiles of Alzheimer disease and frontotemporal dementia: A preliminary examination

**DOI:** 10.1017/cts.2017.38

**Published:** 2018-05-10

**Authors:** Joey Annette Contreras, Shannon L. Risacher, Mario Dzemidzic, John D. West, Brenna C. McDonald, Martin R. Farlow, Brandy R. Matthews, Liana G. Apostolova, Jared Brosch, Bernard Ghetti, Joaquin GoÑi

**Affiliations:** Indiana University School of Medicine, Indianapolis, IN, USA

## Abstract

OBJECTIVES/SPECIFIC AIMS: Recent evidence from resting-state fMRI studies have shown that brain network connectivity is altered in patients with neurodegenerative disorders. However, few studies have examined the complete connectivity patterns of these well-reported RSNs using a whole brain approach and how they compare between dementias. Here, we used advanced connectomic approaches to examine the connectivity of RSNs in Alzheimer disease (AD), Frontotemporal dementia (FTD), and age-matched control participants. METHODS/STUDY POPULATION: In total, 44 participants [27 controls (66.4±7.6 years), 13 AD (68.5.63±13.9 years), 4 FTD (59.575±12.2 years)] from an ongoing study at Indiana University School of Medicine were used. Resting-state fMRI data was processed using an in-house pipeline modeled after Power *et al.* (2014). Images were parcellated into 278 regions of interest (ROI) based on Shen *et al.* (2013). Connectivity between each ROI pair was described by Pearson correlation coefficient. Brain regions were grouped into 7 canonical RSNs as described by Yeo *et al*. (2015). Pearson correlation values were then averaged across pairs of ROIs in each network and averaged across individuals in each group. These values were used to determine relative expression of FC in each RSN (intranetwork) and create RSN profiles for each group. RESULTS/ANTICIPATED RESULTS: Our findings support previous literature which shows that limbic networks are disrupted in FTLD participants compared with AD and age-matched controls. In addition, interactions between different RSNs was also examined and a significant difference between controls and AD subjects was found between FP and DMN RSNs. Similarly, previous literature has reported a disruption between executive (frontoparietal) network and default mode network in AD compared with controls. DISCUSSION/SIGNIFICANCE OF IMPACT: Our approach allows us to create profiles that could help compare intranetwork FC in different neurodegenerative diseases. Future work with expanded samples will help us to draw more substantial conclusions regarding differences, if any, in the connectivity patterns between RSNs in various neurodegenerative diseases.

